# Supplementary material for: DEML: Drug Synergy and Interaction Prediction Using Ensemble-Based Multi-Task Learning
Source: Molecules. 2023 Jan 14;28(2):844. doi: 10.3390/molecules28020844 (PMC9861702; doi:10.3390/molecules28020844)
Supplement: Supplementary file 1 [file molecules-28-00844-s001.zip › molecules-2109842-supplementary.pdf]

Supplementary Materials

DEML: Drug Synergy and Interaction Prediction Using Ensemble-Based Multi-Task Learning

Zhongming Wang [1,2,†], Jiahui Dong [3,†], Lianlian Wu [1,2], Chong Dai [4], Jing Wang [5], Yuqi Wen [2], Yixin Zhang [2], Xiaoxi Yang [2], Song He [2,*] and Xiaochen Bo [2,*]

[1] Academy of Medical Engineering and Translational Medicine, Tianjin University, Tianjin 300072, China
[2] Department of Bioinformatics, Institute of Health Service and Transfusion Medicine, Beijing 100850, China
[3] Department of Pharmaceutical Sciences, Institute of Radiation Medicine, Beijing 100850, China
[4] College of Life Science and Technology, Beijing University of Chemical Technology, Beijing 100029, China
[5] School of Medicine, Tsinghua University, Beijing 100084, China
* Correspondence: hesong@bmi.ac.com (S.H.); boxiaoc@bmi.ac.com (X.B.)
† These authors contributed equally to this work.

The Supplementary Materials include 2 Supplementary Figures and 2 Tables

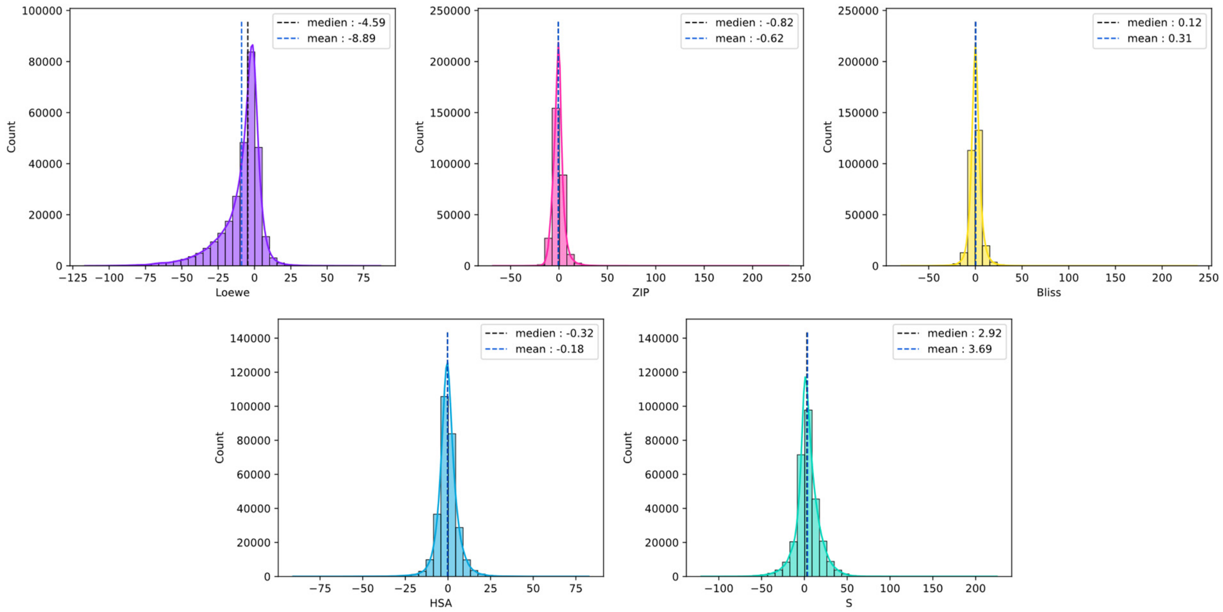

Figure S1. The distribution of different synergy score on dataset.

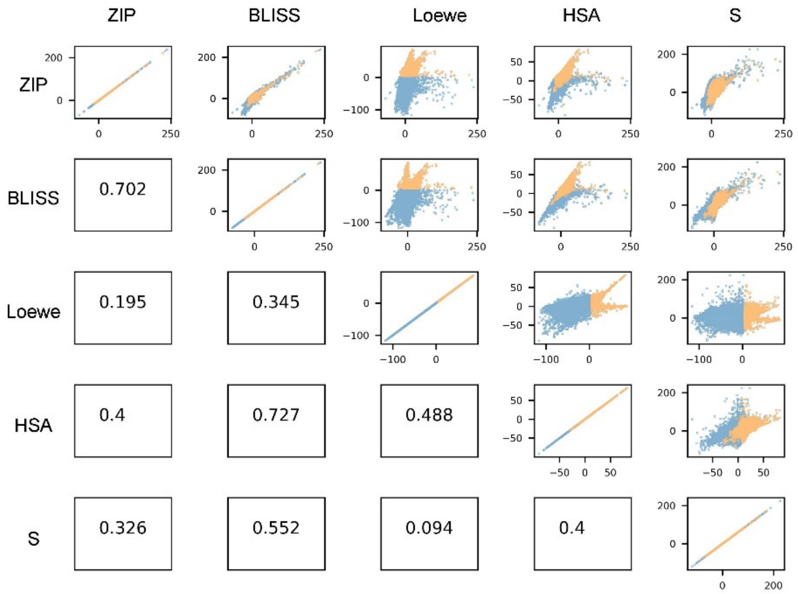

Figure S2. The correlation of different synergy score on dataset. The top right part of figure shows the correlation between different pairs of synergy scores, the point in each subfigure represents a sample with a pair of synergy score. The bottom left subfigures show the Pearson score of the pair of synergy score.

The details of synergy score

The concept of synergy refers to when a combination of drugs has a greater effect than their individual potencies. Generally, synergy is quantified through the properties of dose-response curves, which describe how a drug reacts with specific drugs. Binarized by synergy score using threshold, Drug combinations can be classified as synergism and non-synergism. When the combined effect of two compounds is greater than their single-agent potency, it is considered synergistic combination. When the effect is equal or lower than their single-agent potency, it is considered non-synergistic, including additive type and antagonistic type. There is a variety of synergy quantification model based on different mathematical hypothesis.

the most prominent reference models are Loewe additivity model (Loewe) and Bliss independence model (Bliss), There are also Highest single agent model (HSA), Zero Interaction Potency model (ZIP) and S score. We mainly adopt these five synergy index in regression prediction tasks. We will show the definition of five synergy index as follows.

We define y_c is the inhibition effect produced by drug1 at concentration x_1 combine with drug2 at concentration x_2. $y_1(x_1)$ and $y_2(x_2)$ are their respective single drug effects. The synergy score is calculated as the difference between y_c and the expected effect y_c if there is no synergy. Each synergy scoring took a different model for y_e.

Loewe: y_e is the expected effect of a drug combined with itself, defining

$$S_{Loewe} = y_c - y_1(x_1 + x_2) = y_c - y_2(x_1 + x_2)$$

Bliss: y_e is the expected effect of two drugs acting independently, defining

$$S_{Bliss} = y_c - \left(y_1(x_1) + y_2(x_2) - y_1(x_1)y_2(x_2)\right)$$

HSA: y_e is the maximal single drug effect, defining

$$S_{HSA} = y_c - max\left(y_1(x_1), y_2(x_2)\right)$$

ZIP: y_e is the expected effect of two drugs that do not potentiate each other, defining

$$S_{ZIP} = y_c' - \left(y_1'(x_1) + y_2'(x_2) - y_1'(x_1)y_2'(x_2)\right),$$

$y_1'(x_1)$ and $y_2'(x_2)$ are fitted values for combination and monotherapy drugs based on the full-dose response matrix.

Table S1-1. The division of positive samples for DDI task and the number of each DDI type.

DDI type	Description	Count
	Positive samples of DDI task	Totally 20955
DDI type 26	The risk or severity of adverse effects can be increased when #Drug1 is combined with #Drug2.	7618
DDI type 6	The metabolism of #Drug2 can be decreased when combined with #Drug1.	6019
DDI type 9	The serum concentration of #Drug2 can be decreased when it is combined with #Drug1.	2978
DDI type 18	#Drug1 may increase the cardiotoxic activities of #Drug2.	2771
DDI type 76	#Drug1 may increase the QTc-prolonging activities of #Drug2.	792
DDI type 12	The serum concentration of the active metabolites of #Drug2 can be reduced when #Drug2 is used in combination with #Drug1 resulting in a loss in efficacy.	184
DDI type 70	#Drug1 may increase the myelosuppressive activities of #Drug2.	149
DDI type 52	#Drug1 may increase the bradycardic activities of #Drug2.	121
DDI type 23	#Drug1 may increase the ototoxic activities of #Drug2.	54
DDI type 51	#Drug1 may increase the atrioventricular blocking (AV block) activities of #Drug2.	53
DDI type 48	#Drug1 may increase the antiplatelet activities of #Drug2.	53
DDI type 32	The risk or severity of QTc prolongation can be increased when #Drug1 is combined with #Drug2.	52
DDI type 63	#Drug1 may increase the hypocalcemic activities of #Drug2.	52
DDI type 13	The therapeutic efficacy of #Drug2 can be decreased when used in combination with #Drug1.	29
DDI type 64	#Drug1 may increase the hypoglycemic activities of #Drug2.	27
DDI type 69	#Drug1 may increase the immunosuppressive activities of #Drug2.	3

Table S1-2. The division of negative samples for DDI task and the number of each DDI type.

DDI type	Description	Count
	Negtive Samples of DDI task	Totally 265466
DDI type None	The DDI is not reported by DrugBank database between #Drug1 and #Drug2	260885
DDI type 10	The serum concentration of #Drug2 can be increased when it is combined with #Drug1.	4524
DDI type 15	#Drug1 may decrease the excretion rate of #Drug2 which could result in a higher serum level.	53
DDI type 7	The metabolism of #Drug2 can be increased when combined with #Drug1.	3
DDI type 17	#Drug1 may decrease the cardiotoxic activities of #Drug2.	1

Table S2-1. The hyperparameters settings of XGboost. Note that the bold number is the tuned setting which shows best performance.

Xgboost	Hyperparameter Settings
Number of estimators	50, 100, 200, **500**, 1000
Maximum tree depth	4, **6**, 8, 10, 12
Learning rate	0.01, 0.05, **0.1**, 0.2, 0.5

Table S2-2. The hyperparameters settings of Random Forest. Note that the bold number is the tuned setting which shows best performance.

Random Forest	Hyperparameter Settings
Number of trees	50, 100, **200**, 500, 1000
Maximum tree depth	4, **6**, 8, 10, 12
